# Machine learning for Alzheimer’s disease progression under extreme class imbalance

**DOI:** 10.3389/fnins.2026.1736992

**Published:** 2026-05-25

**Authors:** Patrick O. Akinwumi, Meihua Qian, Taiwo A. Olorunsogbon, Chaoyi Zhou, Siyu Huang

**Affiliations:** 1 College of Education, Clemson University, Clemson, SC, United States; 2 University of Bradford, Bradford, United Kingdom; 3 School of Computing, Clemson University, Clemson, United Kingdom

**Keywords:** Alzheimer’s disease, cognitive decline, explainable AI, longitudinal biomarkers, machine learning in healthcare, XGBoost, logistics regression

## Abstract

**Background:**

Timely identification of individuals at risk for Alzheimer’s disease (AD) progression remains a major clinical challenge. Traditional cognitive assessments provide limited prognostic insight, while many machine learning (ML) models rely on costly biomarkers or poorly interpretable algorithms that limit clinical scalability. This study evaluated whether widely available baseline demographic, clinical, and cognitive measures could support short-term progression prediction using interpretable ML methods under extreme class imbalance.

**Methods:**

We analyzed 3,240 participants from the Alzheimer’s Disease Neuroimaging Initiative (ADNI), of whom 2,423 had valid 24-month follow-up data. The primary outcome was strict unidirectional diagnostic worsening within 24 months (13 events; 0.5%). Baseline demographic, clinical, and cognitive variables were used to train XGBoost and logistic regression models under natural class imbalance using stratified k-fold cross-validation with out-of-fold predictions. Model performance was evaluated using AUROC, area under the precision-recall curve (AUPRC), calibration analyses, and bootstrap confidence intervals. Sensitivity analyses evaluated cost-sensitive learning, threshold optimization, and alternative imputation strategies (KNN and MICE). Longitudinal mixed-effects modeling was conducted separately to characterize cognitive decline and was not used as input to the predictive models. SHAP (Shapley Additive Explanations) quantified feature contributions.

**Results:**

Under natural class imbalance, XGBoost achieved AUROC = 0.912 and AUPRC = 0.051, while logistic regression achieved AUROC = 0.787 and AUPRC = 0.038. Although discrimination exceeded baseline prevalence, precision remained low and threshold optimization produced substantial false-positive burdens, limiting immediate clinical applicability. Cost-sensitive learning did not materially improve performance. MICE imputation produced results comparable to median imputation, whereas KNN imputation reduced performance. SHAP analyses identified baseline cognitive severity, functional measures, and diagnostic status as dominant predictors. Mixed-effects modeling confirmed significant cognitive decline over time (*β* = −0.027 points/month, *p* < 0.001).

**Conclusion:**

Accessible baseline clinical and cognitive variables contain measurable but limited predictive signal for short-term AD progression under extreme event scarcity. These findings should be interpreted as an early-stage proof-of-concept rather than a clinically deployable decision-support tool. External validation remains necessary before clinical translation.

## 1 Introduction

Alzheimer’s disease (AD) is progressive neurodegenerative disorder marked by the accumulation of amyloid-*β* plaques and tau neurofibrillary tangles that begin years before the onset of clinical symptoms, leading to synaptic dysfunction, neuronal loss and eventual dementia (d'Errico and Meyer-Luehmann, 2020; Hampel et al., 2021). AD imposes an enormous health and economic burden: in the US alone, care costs were estimated at US $321 billion in 2022 and are projected to exceed US $1 trillion annually by 2050; globally, dementia-related economic losses may reach US $14.5 trillion by 2050 (Finney et al., 2025; Skaria, 2022), with the likelihood of up to 18.9 million patients in Europe (Canevelli et al., 2019), and 36.5 million in East Asian countries (Prince et al., 2015). Additionally, the global incidence and prevalence of AD and related dementias increased ~150% between 1990 and 2019, making it one of the fastest-rising causes of death and disability worldwide (Calabrò et al., 2020; Li et al., 2022). Early identification of individuals at risk is therefore essential for timely intervention and disease modification (Cullen et al., 2021). To this end, researchers have developed a wide range of biomarkers, including fluid measures, cognitive tests, and genetic profiles, to capture the prodromal stages of AD pathology. Structural MRI, neuropsychological assessments such as the ADAS-Cog and MMSE, and genetic variants each provide complementary diagnostic information (Mattsson et al., 2015; Vemuri and Jack Jr, 2010).

However, traditional dependence on high-cost modalities such as PET and CSF sampling, as well as single-modality approaches, constrains both scalability and predictive accessibility. In parallel, although recent advances in machine learning (ML) and artificial intelligence (AI) have shown promise in Alzheimer’s disease prediction, many existing models rely heavily on costly or invasive biomarkers and often lack interpretability, limiting clinical translation (Jahan et al., 2023). Many ML frameworks remain opaque “black boxes,” offering limited transparency in how predictions are generated. These challenges have motivated growing interest in interpretable machine-learning frameworks that prioritize widely available clinical data while maintaining biological plausibility. To address these gaps, we developed an interpretable machine-learning framework centered primarily on accessible baseline demographic, clinical, and cognitive assessments to model short-term (24-month) clinical progression in AD. Exploratory biomarker analyses were conducted in smaller subsets but were not central to the primary predictive framework. Our approach emphasizes transparent feature attribution, methodological rigor under extreme class imbalance, and realistic interpretation of predictive performance in Alzheimer’s disease research.

## 2 Literature review

Alzheimer’s disease (AD) research has increasingly focused on identifying biological signatures that precede clinical onset. The classical biomarker framework, encompassing amyloid-*β* (Aβ), tau, and neurodegeneration (the A/T/N model), has provided a conceptual foundation for understanding disease progression (Jack Jr et al., 2018). Breakthroughs in molecular medicine have placed the amyloid-β pathway at the center of AD pathophysiology. Although the precise molecular mechanisms and spatiotemporal dynamics leading to synaptic failure and neurodegeneration remain under investigation, established biochemical alterations in the Aβ cycle represent the core biological hallmark of AD and promising therapeutic targets for disease modification.

### 2.1 Plasma and neurochemical biomarkers

Neuroimaging modalities such as PET and MRI offer high diagnostic accuracy but remain costly and resource-intensive, limiting scalability. Consequently, recent studies have emphasized blood-based biomarkers, notably plasma Aβ42/40, phosphorylated tau (p-tau181), glial fibrillary acidic protein (GFAP), and neurofilament light (NfL), which show strong correlations with traditional PET and CSF measures (Palmqvist et al., 2020). Advances in assay technology now allow precise quantification of these plasma “ATN” markers, which are less invasive, more scalable, and cost-effective than cerebrospinal fluid (CSF) and imaging (magnetic resonance and positron emission tomography (PET) approaches (Cullen et al., 2021).

Cullen et al. (2021) demonstrated that combining plasma Aβ42/Aβ40, p-tau217, and NfL with basic demographics significantly improved prediction of cognitive decline in cognitively unimpaired older adults (R^2^ = 0.14) and future AD dementia (AUC = 0.82).^1^ They further estimated that this biomarker combination could reduce clinical trial sample sizes by ~70% while maintaining statistical power. Similarly, Ren et al. (2025) reported that machine learning (ML) models integrating blood biomarkers with demographic and lifestyle factors outperformed MMSE alone in forecasting cognitive decline (internal-test accuracy ~88%). SHAP analysis in that study identified daily living activities, age, and baseline MMSE as top predictors, illustrating the interpretive value of integrating fluid and clinical data.

Despite these advances, plasma biomarkers have important limitations. Their concentrations can be influenced by non-neurological factors (e.g., body mass index and renal function) and may not fully capture regional tau pathology (Vogel et al., 2021; Young et al., 2022). Thus, current plasma assays are not yet suitable as standalone diagnostic tools, and many experts advocate for multimodal integration to improve disease staging and prognostic accuracy (Jasodanand et al., 2025). However, most existing work on blood-based markers has focused primarily on diagnostic classification rather than prospective progression prediction. In the present study, plasma biomarkers were explored in secondary analyses where data were available, but the primary predictive framework prioritized broadly accessible clinical and cognitive measures due to greater availability and stronger practical scalability.

### 2.2 Cognitive and clinical assessments

Neuropsychological testing remains a cornerstone of AD evaluation, providing quantitative measures of memory, executive function, and activities of daily living. Standardized instruments such as the Alzheimer’s Disease Assessment Scale-Cognitive Subscale (ADAS-Cog), Clinical Dementia Rating (CDR), and Mini-Mental State Examination (MMSE) are widely used for both diagnosis and disease staging. Beyond their clinical role, these cognitive scores frequently emerge as top predictive features in data-driven models of AD. For example, Yi et al. (2023) applied an XGBoost framework for multiclass AD classification and identified CDR-Sum-of-Boxes, ADAS-13, and word recall tests as the three most influential features, noting that higher impairment scores were strongly associated with increased AD risk (bmcmedinformdecismak.biomedcentral.com). Similarly, Wang et al. (2024) emphasized that clinical assessments and neuropsychological tests are “crucial” for AD diagnosis and that their integration with biological biomarkers can enhance early detection.

Given that cognitive decline represents the central clinical manifestation of AD, cognitive assessments remain highly informative for risk stratification. Prior work has shown that baseline MMSE, functional measures, and daily living assessments retain strong prognostic value when paired with machine learning approaches (Ren et al., 2025). Consistent with this evidence, our study prioritizes baseline cognitive and functional measures as primary predictive inputs. Longitudinal cognitive modeling was performed separately to characterize population-level decline and inter-individual variability but was not incorporated directly into the machine-learning classifiers.

### 2.3 Genetic and genomic biomarkers

Genetic factors play a major role in AD susceptibility and progression. The ε4 allele of the apolipoprotein E (APOE) gene remains the strongest known genetic risk factor for late-onset AD, while polygenic risk scores (PRS) that aggregate multiple risk variants have further improved the prediction of disease status (Bracher-Smith et al., 2025). However, traditional PRS rely on additive genetic models and may overlook higher-order interactions among loci. Recent machine learning approaches have begun to address this limitation by modeling complex, nonlinear patterns in genome-wide data (Bracher-Smith et al., 2025).

Mirabnahrazam et al. (2022) developed a “dementia score” combining MRI and genetic features to predict conversion to AD. Their results showed that genetic data alone achieved higher predictive accuracy in cognitively normal individuals (~0.857) compared with MRI alone (~0.143), while MRI better characterized subjects with stable MCI. Importantly, combining genetic and imaging features yielded the best performance in intermediate-risk cases (Mirabnahrazam et al., 2022), suggesting that genetic information may signal risk long before neurodegenerative changes are anatomically detectable, whereas imaging captures later structural progression.

In line with this evidence, our study incorporates genetic biomarkers, including APOE ε4 carrier status and polygenic risk indicators, to exploit early, heritable risk signals. By integrating these genetic measures with fluid and cognitive data, we aim to identify at-risk individuals at the preclinical stage. Notably, Bracher-Smith et al. (2025) demonstrated that advanced ML methods, such as gradient boosting and neural networks, can replicate known AD-associated loci and uncover novel variants. While our focus is not gene discovery, these findings highlight the potential of machine learning to integrate genomic complexity into predictive frameworks, an approach that underpins the design of our model.

### 2.4 Neuroimaging biomarkers

Neuroimaging offers direct insights into brain structure and function and remains a cornerstone of AD research. Structural MRI, assessing hippocampal and cortical volumes, reflects neurodegenerative atrophy, whereas PET enables *in vivo* visualization of amyloid and tau deposition, both integral components of the A/T/N biomarker framework. Despite their diagnostic value, these modalities face important practical limitations: PET imaging is costly and not widely accessible, and conventional MRI can be insensitive to very early pathological changes.

Empirical evidence suggests that when imaging data are integrated with other modalities, their relative predictive weight often diminishes. Yi et al. (2023) found that among numerous imaging-derived variables, only ventricular volume entered the final predictive model, and even this contributed minimally. Their interpretable ML framework modestly prioritized ventricular size but showed that cognitive test scores dominated model importance. These findings indicate that, by themselves, common MRI measures may be less informative for early-stage AD classification than cognitive or fluid biomarkers.

Nevertheless, neuroimaging remains an important complementary modality in Alzheimer’s disease research. Structural MRI can delineate atrophy patterns associated with disease stage (Mirabnahrazam et al., 2022), while PET imaging continues to support disease staging and clinical trial enrollment. However, these modalities remain costly and less scalable in routine care settings. Because the primary goal of this study was to evaluate prediction using accessible, low-cost structured data, neuroimaging variables were not included in the primary predictive framework.

### 2.5 Machine learning and AI techniques

Machine learning, a subfield of artificial intelligence (AI) that enables algorithms to learn patterns from data and improve performance through experience, has become indispensable in Alzheimer’s disease research for integrating heterogeneous and high-dimensional biomarker data. Among ML methods, tree-based ensemble algorithms such as XGBoost and Random Forests have shown consistently high classification accuracy in AD prediction tasks. In our framework, we employ XGBoost, which effectively handles heterogeneous inputs, missing data, and class imbalance (Yi et al., 2023). Comparative studies have demonstrated that XGBoost frequently outperforms traditional classifiers. For instance, Jahan et al. (2023) evaluated nine ML algorithms (including Random Forest, SVM, and boosting methods) on a multimodal AD dataset and reported that Random Forest achieved the best accuracy (98.8%). Similar to these efforts, we benchmarked XGBoost against logistic regression to compare predictive performance under extreme class imbalance while preserving interpretability.

A major methodological advance in recent AD modeling has been the integration of explainable AI (XAI) tools. The SHAP (SHapley Additive exPlanations) framework (Nohara et al., 2022) enables feature-level attribution, transforming traditionally opaque ML models into interpretable, clinically meaningful tools. Yi et al. (2023) explicitly combined SHAP with XGBoost to construct an interpretable AD classifier, showing how SHAP values reveal both the direction and magnitude of each biomarker’s effect on disease risk. Similarly, Ren et al. (2025) used SHAP to dissect their cognitive-decline model, identifying daily living activities, age, and baseline MMSE as dominant predictors. By incorporating SHAP in our study, we enable clinicians to understand and verify model decisions, thereby enhancing trust, interpretability, and adoption in clinical contexts.

Deep learning approaches, such as neural networks, have also been applied to AD, particularly for imaging and multimodal data fusion. For example, Wang et al. (2024) developed a deep network integrating structural MRI, clinical, and genetic data, achieving a high AUC (~0.96) for 4-year MCI-AD conversion prediction While deep models capture complex nonlinear relationships, they often face challenges such as limited interpretability and data scarcity in biomedical settings (Choi et al., 2018; Lian et al., 2020; Zhu et al., 2021). Consequently, our study leverages XGBoost for its balance between predictive power and transparency, combining feature selection and boosting to model heterogeneous biomarkers effectively. We adopt established strategies, such as class reweighting to address imbalance (Yi et al., 2023), and use SHAP to distill the model into an optimal, interpretable feature subset. This dual focus on accuracy and interpretability aligns with the growing consensus that explainable AI is essential for trustworthy healthcare applications (Jahan et al., 2023).

### 2.6 The present study

Despite substantial progress in applying machine learning and artificial intelligence to Alzheimer’s disease, important limitations remain in the existing literature. Many prior studies emphasize diagnostic classification rather than prospective progression prediction, frequently rely on costly imaging or biomarker modalities, and often prioritize predictive accuracy over interpretability (Yi et al., 2023). Severe class imbalance remains another under-addressed challenge in Alzheimer’s progression modeling, particularly when clinically meaningful progression events are rare. Many studies also report strong discrimination metrics without adequately discussing calibration, precision-recall tradeoffs, or real-world clinical implications.

To address these challenges, the present study develops an interpretable machine-learning framework using the ADNI dataset to evaluate short-term (24-month) clinical progression under extreme event scarcity. The primary predictive models relied exclusively on baseline demographic, clinical, and cognitive variables that are widely available in routine clinical settings. Logistic regression and XGBoost models were evaluated under the natural outcome distribution using stratified cross-validation, with SHAP used to quantify feature contributions and assess biological plausibility. Longitudinal mixed-effects modeling was performed separately to characterize cognitive decline trajectories but was not used as a predictive input. Additional sensitivity analyses evaluated alternative missing-data strategies (median imputation, K-nearest neighbors, and multiple imputation by chained equations (MICE), cost-sensitive learning approaches, threshold optimization, and exploratory biomarker subsets to assess methodological robustness under severe imbalance. Given the very small number of progression events, this study is intentionally framed as an early-stage proof-of-concept designed to assess whether accessible baseline clinical data contain measurable, but limited, predictive signal for short-term Alzheimer’s disease progression rather than as a clinically deployable decision-support system. External validation in independent cohorts remains necessary to determine whether these findings generalize beyond ADNI.

## 3 Methods

### 3.1 Data source and cohort

Data was obtained from the Alzheimer’s Disease Neuroimaging Initiative (ADNI; phases ADNI-1, GO, 2, and 3), a multicenter longitudinal study designed to characterize Alzheimer’s disease (AD) progression and validate biomarkers across the cognitive spectrum (http://adni.loni.usc.edu). We integrated harmonized clinical, neuropsychological, genetic, and fluid biomarker data (snapshot: October 13, 2025) from PTDEMOG, DXSUM, MMSE, MOCA, ADAS, BLCHANGE, ADSXLIST, APOERES, UPENN_PLASMA_FUJIREBIO_QUANTERIX, and UPENNMIRNA. Longitudinal records were aligned per subject using RID and standardized visit dates (VISCODE/EXAMDATE). A total of 3,801 participant-level observations remained after dataset merging, exclusion of variables with >50% missingness, and removal of records with unresolved missing values following initial preprocessing. After exclusion of individuals lacking complete baseline demographic or diagnostic data, the analytic dataset comprised 3,240 participants. Of these, 2,423 participants had valid diagnostic follow-up within 24 months and constituted the final modeling cohort. All predictors used in the primary models were derived exclusively from the baseline visit. Administrative identifiers (e.g., subject ID, duplicate ID fields, SITEID) were excluded from the final feature set to prevent site- or dataset-specific artifacts from influencing prediction.

The primary endpoint (PROG_24M) was defined as strict unidirectional diagnostic worsening within 24 months, limited to transitions from cognitively normal (CN) to mild cognitive impairment (MCI), MCI to AD, or CN directly to AD. Diagnostic reversion (e.g., MCI → CN) and fluctuating classifications were not considered progression events. Participants were required to have a defined baseline diagnosis and at least one follow-up assessment within 24 months. Sentinel missing codes were harmonized prior to analysis. Feature completeness was evaluated before modeling; plasma NfL (NfL_Z) exhibited complete availability, whereas longitudinal slope features (e.g., MMSCORE_SLOPE_YR) showed >99% missingness in the strict 24-month cohort. Accordingly, slope-derived variables were not retained in the primary imbalanced models, and baseline predictors constituted the principal analytic framework.

### 3.2 Outcomes

The primary endpoint (PROG_24M) was defined as unidirectional diagnostic worsening within 24 months using longitudinal diagnosis codes from ADNI DXSUM. For each participant (RID), all visits with non-missing diagnosis values were chronologically ordered by visit month (VIS_MO), and the earliest available diagnosis was designated as the baseline diagnostic state (*t* = 0). Follow-up visits eligible for endpoint determination were those occurring after baseline and within 24 months (0 < VIS_MO ≤ 24). Participants without any eligible follow-up visits in this window were assigned missing endpoint status and excluded from the progression modeling cohort.

Diagnostic severity was treated ordinally (CN < MCI < AD). A participant was labeled a progressor (PROG_24M = 1) if a worsening transition (CN → MCI/AD or MCI → AD) occurred within the 24-month window and the diagnosis did not subsequently revert to a less severe category within that same window (i.e., no MCI → CN or AD→MCI/CN after the first worsening). Participants with no worsening transitions within 24 months, or with diagnostic fluctuation that included reversion after worsening, were labeled as non-progressors (PROG_24M = 0). In the final modeling cohort (*n* = 2,423), 13 individuals (0.5%) met criteria for 24-month unidirectional worsening (0.29% among baseline CN; 0.50% among baseline MCI; 0.00% among baseline AD).

To ensure reproducibility, the label-generation procedure is summarized in pseudocode below.

For each participant i:

1) Collect all DXSUM visits with non-missing DIAGNOSIS.

2) Sort visits by VIS_MO ascending.

3) Define baseline:

t0 = earliest VIS_MO.

d0 = DIAGNOSIS at t0.

4) Define eligible follow-up window:

FU = visits with (VIS_MO > t0) and (VIS_MO ≤ t0 + 24).

If FU is empty: PROG_24M = missing (exclude from modeling).

5) Identify first worsening within window:

Find earliest visit in FU where DIAGNOSIS > d0.

If none exists: PROG_24M = 0.

6) Fluctuation handling (unidirectional requirement):

Let t* be time of first worsening and d* its DIAGNOSIS value.

If any later visit in FU with VIS_MO ≥ t* has DIAGNOSIS < d*:

PROG_24M = 0 (reversion/fluctuation).

Else:

PROG_24M = 1 (unidirectional worsening).

### 3.3 Feature engineering

Demographic, clinical, and visit-level variables were harmonized by merging tables on RID and VISCODE (or RID when VISCODE was unavailable), with date fields standardized to construct per-subject timelines. Baseline features were defined using the first available diagnostic visit for each participant. Variables derived from demographic data included age proxies and education. APOE ε4 carrier status (APOE4_CARRIER) was derived from APOERES genotype information (E2/E3/E4) and encoded as a binary indicator (carrier vs. non-carrier). Due to incomplete APOE coverage in the final modeling cohort, APOE interaction analyses were evaluated only exploratorily and were not retained in the primary models.

### 3.4 Plasma biomarkers, miRNA integration, and data handling

Plasma biomarkers were extracted from the UPENN_PLASMA_FUJIREBIO_QUANTERIX dataset. Columns corresponding to Aβ42, Aβ40, phosphorylated tau species (e.g., p-tau181, p-tau217), GFAP, and neurofilament light (NfL) were programmatically identified. Derived ratio features (e.g., Aβ42/40, p-tau/Aβ42, p-tau/Aβ40, GFAP/NfL) were computed where available, and log-transformations were applied to reduce skewness and stabilize variance. These plasma markers have been extensively characterized in the AD biomarker literature as reflecting amyloid deposition, tau pathology, and neurodegeneration (Jack et al., 2008).

Numeric miRNA features from UPENNMIRNA were standardized prior to modeling. When dimensionality permitted, principal component analysis (PCA) was applied to summarize high-dimensional miRNA profiles into orthogonal components (miRNA_PCs), reducing multicollinearity and improving numerical stability. To explore cross-omic coordination, we computed a miRNA-Proteome Coupling Index (MPCI) for subjects with overlapping plasma and miRNA data. MPCI was defined as the mean absolute partial correlation between miRNA principal components and plasma biomarkers, estimated using a shrinkage-based covariance inversion approach. When longitudinal samples were available, nearest-date matching within ±90 days was applied; otherwise, first-available samples were used. Due to limited overlap (approximately 52 subjects within the final modeling cohort), MPCI analyses were treated as exploratory feasibility assessments and were not central to the primary predictive models.

Variables with >50% missingness were excluded prior to modeling. ADNI sentinel codes were treated as missing values. Remaining missing data were initially handled using median imputation for the primary analyses to preserve reproducibility under extreme event scarcity. Sensitivity analyses additionally evaluated K-nearest neighbors (KNN) imputation and multiple imputation by chained equations (MICE) to assess whether alternative imputation strategies materially altered model performance. These alternative approaches produced comparable results and did not substantially change model discrimination. Per-subject omics missingness rates were additionally computed to assess potential missingness-as-signal effects; however, these features did not materially alter model discrimination in the final analysis.

### 3.5 Predictive modeling

The primary modeling task was to predict 24-month unidirectional clinical progression (PROG_24M) using baseline-derived numeric features encompassing demographic, diagnostic, cognitive, functional, and available biomarker variables, although the primary predictive signal was expected to derive from baseline clinical and cognitive features. Longitudinal slope features were excluded from the primary imbalanced analysis due to substantial missingness in the strict 24-month cohort. Administrative identifiers were not included as predictors.

Two model classes were evaluated: (1) regularized logistic regression and (2) gradient-boosted decision trees implemented using XGBoost (Chen and Guestrin, 2016). Both models were trained using identical baseline feature sets to enable direct comparison between linear and nonlinear approaches. XGBoost was configured with conservative hyperparameters (n_estimators = 400, max_depth = 4, learning_rate = 0.05, subsample = 0.8, colsample_bytree = 0.8) to capture nonlinear relationships and interaction effects while limiting overfitting. Logistic regression was implemented with L2 regularization.

Model evaluation was conducted using stratified 5-fold cross-validation, with performance summarized using out-of-fold predictions. Given the extreme class imbalance in the final modeling cohort (13 progressors; 0.5%), discrimination and calibration were assessed using the area under the receiver operating characteristic curve (AUROC; DeLong et al., 1988), area under the precision-recall curve (AUPRC), and Brier score (Glenn, 1950). Threshold operating characteristics were examined using Youden’s J statistic and F1 score on pooled out-of-fold predictions to illustrate sensitivity-specificity trade-offs under real-world prevalence.

Oversampling approaches were examined as a sensitivity analysis to assess whether balancing class distributions altered model performance. Preliminary sensitivity explored oversampling approaches for class imbalance; however, these analyses were not retained in the final evaluation because synthetic resampling can produce unstable and potentially misleading estimates when only 13 progression events are available. Accordingly, primary analyses focused on preserving the natural outcome distribution and evaluating algorithm-level strategies such as class weighting and threshold optimization. To address severe outcome imbalance without altering the underlying data distribution, sensitivity analyses additionally evaluated cost-sensitive learning through inverse class weighting in both logistic regression and XGBoost models.

Model interpretability was assessed using SHapley Additive exPlanations (SHAP) values for XGBoost models (Lundberg and Lee, 2017), and standardized coefficient magnitudes for logistic regression.

#### 3.5.1 Model evaluation

To rigorously assess model performance, we selected metrics that jointly capture discriminative power, calibration, and clinical utility. The area under the receiver operating characteristic curve (AUROC) quantified overall discrimination between progressors and non-progressors, providing a threshold-independent measure of separability commonly used in medical prognosis modeling (Bron et al., 2021). The area under the precision–recall curve (AUPRC) complemented AUROC by emphasizing performance in the positive (progressor) class, which is clinically more consequential given class imbalance (Saito and Rehmsmeier, 2015). Calibration was evaluated using the Brier score and reliability plots to ensure probabilistic outputs aligned with observed event rates, as recommended for clinical risk models (Niculescu-Mizil and Caruana, 2005). Threshold-based summaries, including sensitivity, specificity, positive predictive value (PPV), and negative predictive value (NPV), were derived from the Youden and F1-optimal cutoffs to support interpretability in real-world diagnostic contexts. These metrics were selected to provide a balanced assessment of discrimination, reliability, and potential research utility under extreme outcome imbalance.

AUROC (Area Under the Receiver Operating Characteristic Curve): Measures the model’s ability to distinguish between classes across all classification thresholds, represent as:

$$\text{AUROC}=\int_{0}^{1}\mathrm{TPR}(\mathrm{EPR})d(\mathrm{EPR})$$

AUPRC (Area Under the Precision-Recall Curve) evaluates a model’s performance on the positive class and is especially relevant for imbalanced datasets. It is calculated as:

$$\text{AUPRC}=\int_{0}^{1}\text{Precision}(\text{Recall})d(\text{Recall})$$

Brier Score—Quantifies the mean squared difference between predicted probabilities and actual binary outcomes. It is expressed below:

$$\text{Brier score}=\frac{1}{N}\sum_{i=1}^{N}{\left({\hat{P}}_{i}-y_{i}\right)}^{2}$$

Sensitivity (Recall)—measures the proportion of true positives correctly identified among all actual positives, calculated as follows:

$$\text{Sensitivity}/\text{Recall}=\frac{\mathrm{TP}}{\mathrm{TP}+\mathrm{FN}}$$

Specificity—Proportion of true negatives correctly identified among all actual negatives, calculated as:

$$\text{Specificity}=\frac{\mathrm{TN}}{\mathrm{TN}+\mathrm{FP}}$$

Positive Predictive Value (Precision) is the proportion of true positives among all predicted positives, calculated as:

$$\text{Precsion}=\frac{\mathrm{TP}}{\mathrm{TP}+\mathrm{FP}}$$

F1 Score—Harmonic mean of precision and recall, balancing both in a single metric, expressed as:

$$F-\text{score}=2*\frac{\text{Precision}*\text{Recall}}{\text{Precision}+\text{Recall}}$$

Youden’s J Index quantifies a diagnostic test’s performance by maximizing the combined value of its sensitivity and specificity. It is calculated as:

J=Sensitivity+specificty−1

### 3.6 Explainability and feature relevance

Feature relevance was assessed using two complementary approaches: permutation importance and SHapley Additive exPlanations (SHAP). Permutation importance was computed by measuring performance degradation following random shuffling of individual features, providing a model-agnostic estimate of variable contribution based on impact on predictive discrimination.

For XGBoost models, SHAP values were computed using the tree-based explainer (Fisher et al., 2019; Lundberg and Lee, 2017) to quantify both global and individual-level feature contributions on the probability scale. SHAP values enable consistent attribution of model predictions by decomposing outputs into additive feature contributions derived from cooperative game theory. For logistic regression, standardized coefficient magnitudes were examined to assess direction and relative strength of associations. Feature relevance and SHAP patterns were evaluated across cross-validation folds to ensure stability of dominant predictors.

### 3.7 Statistical tests and sensitivity analyses

In exploratory subgroup analyses involving limited sample sizes (e.g., the MPCI cohort), distributional differences were evaluated using the Mann–Whitney U test, and effect sizes were quantified using Cliff’s *δ* as a nonparametric measure of magnitude (Cliff, 1993; Molnar et al., 2024; Niculescu-Mizil and Caruana, 2005; Vargha and Delaney, 2000). These analyses were exploratory and not part of the primary predictive modeling framework.

To assess methodological robustness under extreme event scarcity, additional sensitivity analyses were conducted using cost-sensitive learning, threshold optimization, and alternative missing-data approaches. Class-weighted logistic regression and XGBoost models were evaluated to determine whether algorithm-level weighting improved minority class detection. Threshold optimization analyses were performed using F1-based decision thresholds to assess precision-recall tradeoffs under low-prevalence conditions. Missing-data sensitivity analyses were also conducted using K-nearest neighbor (KNN) imputation and Multiple Imputation by Chained Equations (MICE) to evaluate whether predictive performance was sensitive to the original median imputation strategy.

### 3.8 Computing environment

All analyses were conducted in Python, using pandas, scikit-learn, xgboost, statsmodels, and shap libraries. Data preprocessing pipelines incorporated internal imputation and scaling steps to prevent information leakage between training and validation phases.

## 4 Results

### 4.1 Cohort overview

After harmonization and merging of ADNI tables, 3,801 participants with valid baseline diagnostic information were initially identified. Following baseline completeness filtering, the analytic dataset comprised 3,240 participants. Among these, 2,423 individuals had valid diagnostic follow-up within 24 months and constituted the final modeling cohort.

Within this cohort, 13 participants (0.5%) met criteria for strict unidirectional diagnostic worsening within 24 months (CN → MCI, MCI → AD, or CN → AD). Transition rates by baseline group were low: 0.29% among baseline cognitively normal participants, 0.50% among baseline mild cognitive impairment participants, and 0.00% among baseline AD participants. This distribution reflects the extreme class imbalance characteristic of short-term progression in ADNI.

Longitudinal cognitive slope features were evaluated; however, in the strictly defined 24-month cohort, slope completeness was limited (>99% missing for MMSCORE_SLOPE_YR), and these variables were therefore excluded from the primary predictive models. Mixed-effects modeling conducted at the visit level confirmed a modest but statistically significant average decline in MoCA over time (*β* ~ −0.30 points per year, *p* < 0.001), consistent with expected longitudinal cognitive trajectories in ADNI; however, these longitudinal parameters were not used as primary predictors in the final imbalanced classification models.

#### 4.1.1 Plasma and miRNA coverage

Plasma biomarkers (Aβ42, Aβ40, phosphorylated tau species, GFAP, and NfL) were available for the majority of participants in the analytic dataset, whereas miRNA expression data were available for a smaller subset. Within the final 24-month modeling cohort (*n* = 2,423), cross-omic overlap sufficient to compute the miRNA–Proteome Coupling Index (MPCI) was present for 52 participants. Due to this limited coverage, MPCI analyses were treated as exploratory feasibility assessments and were not central to the primary predictive models.

### 4.2 Model performance and calibration (primary analysis: real-world imbalanced 24-month cohort)

Under stratified 5-fold cross-validation on the final 24-month modeling cohort (*n* = 2,423; 13 progressors, 0.5%), XGBoost achieved a mean AUROC of approximately 0.912 and a mean AUPRC of approximately 0.05 (see Table 1). Logistic regression trained on the identical baseline feature set demonstrated comparable discrimination. Out-of-fold (OOF) predictions yielded consistent performance, confirming stability of risk ranking across folds. Model performance was evaluated in the strictly defined 24-month modeling cohort (*n* = 2,423), comprising 13 participants (0.5%) meeting criteria for unidirectional diagnostic worsening. Given the extreme class imbalance, performance was assessed using stratified 5-fold cross-validation with out-of-fold prediction aggregation.

**Table 1:** Model performance (imbalanced data).

Fold	AUROC	AUPRC	Brier score
XGBoost (Baseline)	0.912	0.05	0.0055
XGBoost (All)	0.912	0.05	0.0055
Logistic Regression (Baseline)	0.79	0.04	0.0056

#### 4.2.1 Event distribution across cross-validation folds

Because the progression endpoint was rare (13 events), event distribution across the five stratified cross-validation folds was examined. Each validation fold contained 2–3 progression events (Table 2), confirming that events were distributed across folds rather than concentrated within a single partition.

**Table 2:** Distribution of progression events across cross-validation folds (5-fold CV).

Fold	Total samples	Events	Non-events
Fold 1	485	3	482
Fold 2	485	3	482
Fold 3	485	3	482
Fold 4	484	2	482
Fold 5	484	2	482

This distribution highlights the extreme outcome scarcity and the resulting uncertainty in performance estimates.

#### 4.2.2 Model discrimination

The progression endpoint was rare (13/2423 events; 0.5%). Stratified 5-fold cross-validation distributed events across validation folds (2–3 events per fold), confirming that outcome scarcity was consistent across partitions. Under natural class imbalance, XGBoost demonstrated the strongest discrimination, achieving AUROC 0.912 (95% CI 0.837–0.963) and AUPRC 0.051 (95% CI 0.024–0.122). Logistic regression trained on the same baseline feature set achieved AUROC 0.787 (95% CI 0.629–0.924) and AUPRC 0.038 (95% CI 0.011–0.128; Table 3). Inclusion of longitudinal slope variables did not materially alter discrimination (AUROC 0.912; AUPRC 0.051), consistent with their limited availability in the strict progression cohort. XGBoost demonstrated stronger rank-based discrimination than logistic regression under the natural outcome distribution; however, precision-recall performance remained limited due to the extremely low event prevalence (13 events; 0.5%). These findings indicate detectable predictive signal, but performance estimates should be interpreted cautiously given the small number of progression events.

**Table 3:** Model performance (imbalanced data).

Model	AUROC (95% CI)	AUPRC (95% CI)
Logistic Regression (Baseline)	0.787(0.629–0.924)	0.038(0.011–0.128)
XGBoost (Baseline)	0.912(0.837–0.963)	0.051(0.024–0.122)
XGBoost (All)	0.912(0.837–0.963)	0.051(0.024–0.122)

#### 4.2.3 Threshold characteristics

**Figure 1:**
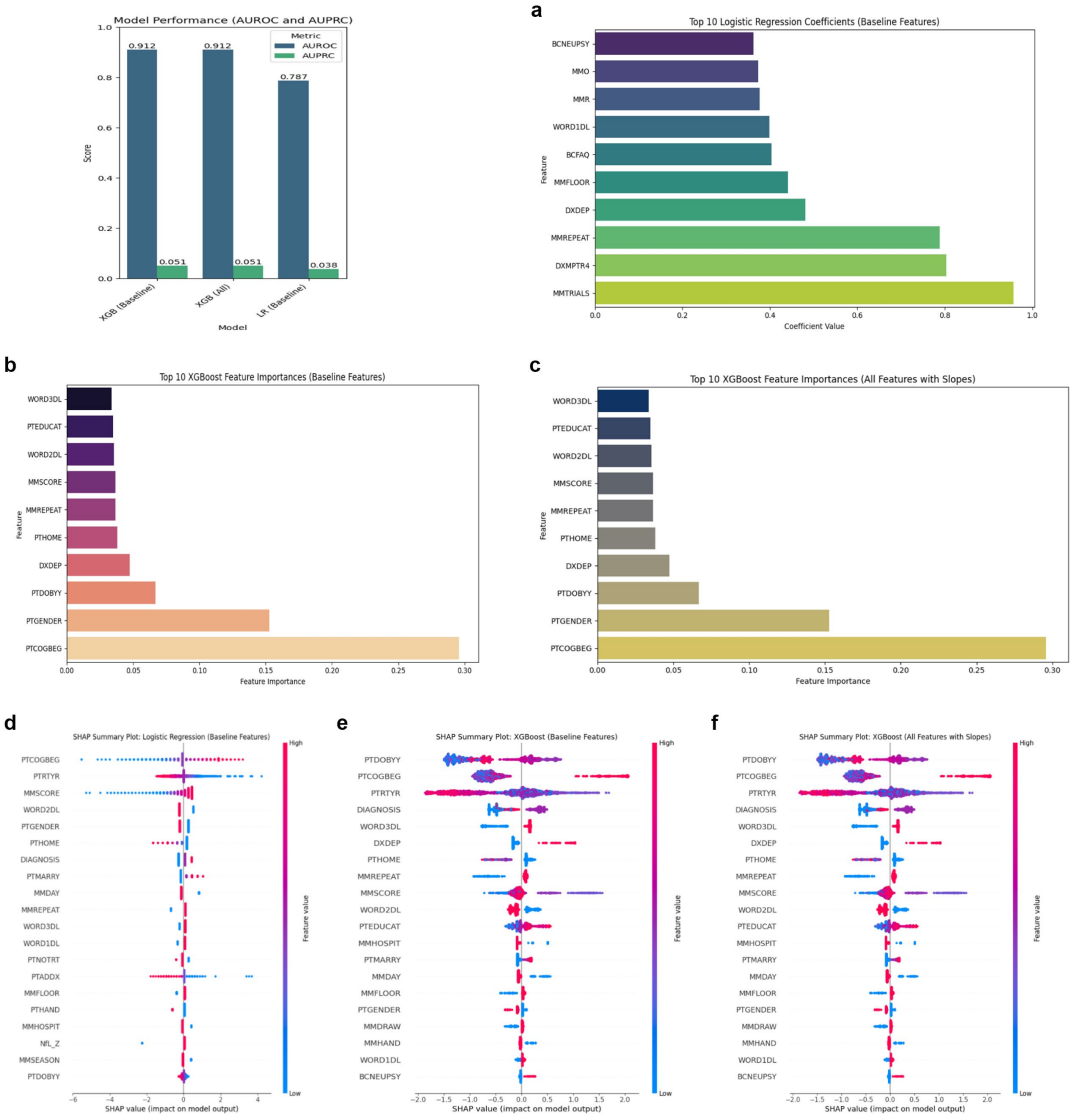
Model performance (imbalanced data). **(a)** Feature importance logistic regression (baseline features). **(b)** Feature importance XGBoost (baseline features). **(c)** Feature importance XGBoost (all features). **(d)** SHAP explainability of LR (baseline features). **(e)** SHAP explainability of XGBoost (baseline features). **(f)** SHAP explainability of XGBoost (all features).

Given the extremely low 24-month progression prevalence (0.5%; Table 2; Figure 1), sensitivity at conventional probability thresholds was inherently constrained. However, the high AUROC (0.912) indicates strong rank-based discrimination, meaning that individuals who progressed were consistently assigned higher predicted probabilities than non-progressors. Although AUPRC (~0.05) exceeded baseline prevalence (~0.005), precision remained low in absolute terms. This indicates that many patients identified as high-risk would represent false positives under real-world deployment conditions. Accordingly, threshold analyses are presented descriptively and should not be interpreted as clinically deployable operating thresholds. Given the extreme class imbalance, precision-recall performance must be interpreted relative to the low baseline prevalence (0.5%). Although sensitivity at default probability thresholds were limited, overall discrimination remained robust. Brier scores were numerically low; however, this largely reflects outcome rarity rather than strong probability calibration. Calibration curves should therefore be interpreted cautiously because most probability bins contained few or no events. Threshold analyses (Table 1) illustrate trade-offs between sensitivity, specificity, and precision, emphasizing that operating points would require external calibration for clinical deployment.

Because the dataset contained only 13 progression events (0.5%), threshold-dependent performance metrics are inherently unstable. In 5-fold cross-validation each validation fold contained only 2–3 events, limiting reliable estimation of sensitivity and precision at specific probability thresholds.

The observed AUPRC (~0.05) was approximately tenfold higher than the baseline prevalence (~0.005), indicating enrichment of true progressors among higher-risk predictions despite the extreme imbalance.

Threshold analyses (Table 3) are presented descriptively to illustrate potential operating trade-offs, but should not be interpreted as stable clinical operating points given the limited number of events.

#### 4.2.4 Sensitivity analyses under extreme class imbalance

To assess robustness under extreme outcome rarity, additional sensitivity analyses evaluated class-weighted learning, threshold optimization, and alternative missing-data strategies.

##### 4.2.4.1 Cost-sensitive learning

Applying inverse-frequency class weighting did not materially improve discrimination relative to the primary models. Weighted logistic regression achieved AUROC = 0.722, AUPRC = 0.037, while weighted XGBoost achieved AUROC = 0.901 and AUPRC = 0.044. These results were comparable to the primary imbalanced models, indicating that algorithm-level weighting alone did not substantially overcome limitations imposed by the small number of progression events.

For algorithm-level imbalance handling, class-weighted logistic regression and cost-sensitive XGBoost were implemented using inverse prevalence weighting (positive class weight = 185.38). These approaches did not materially improve discrimination relative to the primary models. Class-weighted logistic regression achieved AUROC = 0.722 and AUPRC = 0.037, while cost-sensitive XGBoost achieved AUROC = 0.901 and AUPRC = 0.044, compared with AUROC = 0.912 and AUPRC = 0.051 in the primary XGBoost model.

##### 4.2.4.2 Threshold optimization

Threshold optimization demonstrated substantial precision-recall trade-offs. For baseline logistic regression, the F1-optimized threshold yielded precision = 0.087 and recall = 0.308 (4 true positives; 42 false positives). Baseline XGBoost achieved precision = 0.071 and recall = 0.231 (3 true positives; 39 false positives). Weighted models showed similarly unstable operating characteristics. These findings highlight the high false-positive burden associated with deployment under extreme prevalence conditions.

##### 4.2.4.3 Alternative imputation strategies

To evaluate robustness to missing-data assumptions, models were re-estimated using KNN and MICE imputation. KNN imputation reduced discrimination for both logistic regression (AUROC = 0.697, AUPRC = 0.018) and XGBoost (AUROC = 0.876, AUPRC = 0.027). MICE similarly reduced logistic regression performance (AUROC = 0.674, AUPRC = 0.014), while XGBoost performance remained comparable to the primary analysis (AUROC = 0.920, AUPRC = 0.050). These findings suggest that the primary conclusions were robust across alternative preprocessing strategies.

#### 4.2.5 Feature relevance and explainability

Feature importance analyses consistently showed that baseline cognitive severity, functional assessments, and demographic variables were the dominant predictors across both logistic regression and XGBoost models (Figures 1a–c) and SHAP (Figures 1d–f). SHAP analyses identified baseline cognitive status (PTCOGBEG, MMSCORE), diagnostic status, education, and functional measures as the most influential contributors to progression predictions. Plasma biomarker and exploratory omics variables contributed minimally to model performance, reinforcing that the primary predictive signal was driven largely by widely available clinical features. These findings suggest that baseline clinical and cognitive variables contain detectable prognostic signal; however, the current models remain exploratory and are not suitable for clinical deployment without substantially larger event cohorts and external validation.

#### 4.2.6 Calibration

**Figure 2:**
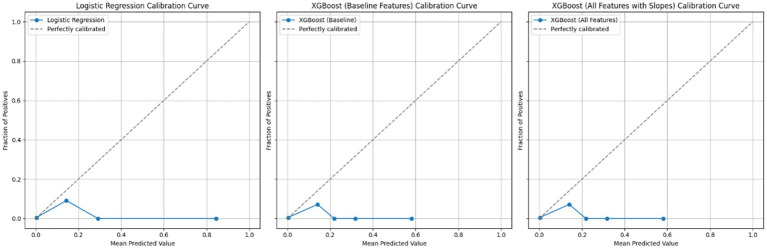
Calibration.

Calibration curves (Figure 2) illustrate the relationship between predicted probabilities and observed event frequencies. Because the dataset contained only 13 progression events, most probability bins contained no events, resulting in substantial variability in the observed fraction of positives. Consequently, calibration estimates, particularly at moderate or higher predicted probabilities, are unstable and should be interpreted cautiously.

Across models, predicted probabilities remained concentrated in low-risk ranges, consistent with the overall event prevalence (0.5%). Although Brier scores were low (0.005), this largely reflects the rarity of the outcome rather than definitive evidence of well-calibrated probabilities. Accordingly, the calibration curves are presented descriptively, and stronger calibration conclusions would require substantially larger numbers of progression events.

Accordingly, discrimination metrics with uncertainty estimates are emphasized, while threshold and calibration analyses are interpreted cautiously given the limited number of observed events.

### 4.3 Longitudinal mixed-effects modeling of cognitive change

To characterize longitudinal cognitive trajectories independently of the classification framework, we fitted a linear mixed-effects model with MMSCORE as the dependent variable, time in months (VIS_MO) as a fixed effect, and a subject-specific random intercept for participant ID (RID). The model was estimated using restricted maximum likelihood (REML) on 2,518 visit-level observations nested within 2,423 participants, allowing separation of population-level cognitive decline from individual baseline variability.

Time was significantly associated with declining cognitive performance (*β* = −0.027 points per month, 95% CI [−0.034, −0.019], *p* < 0.001), corresponding to an average annual decline of approximately 0.32 MMSE points. The fixed intercept (*β* = 27.67, *p* < 0.001) represents the estimated mean baseline MMSCORE at time zero across participants.

**Figure 3:**
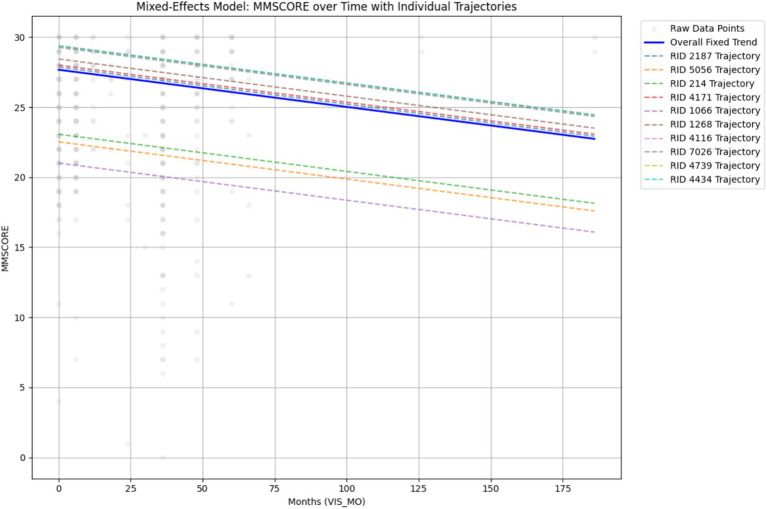
Mixed-effects modeling of cognitive decline plot.

Substantial between-subject heterogeneity in baseline cognition was observed (random intercept variance = 7.36), indicating considerable variability in initial cognitive status. As shown in Figure 3, individual trajectories demonstrated largely parallel downward trends with differing intercepts, consistent with progressive but heterogeneous cognitive decline across participants.

These findings confirm a statistically significant but modest average decline in cognition over time within the ADNI cohort. Importantly, this longitudinal analysis was conducted independently of the machine-learning framework and was used solely to characterize cohort-level cognitive trajectories. Outputs from the mixed-effects model were not included as predictors in the progression classification models, which relied exclusively on baseline demographic, clinical, and cognitive variables (Table 4).

**Table 4:** Linear mixed-effects model of MMSE over time.

Parameter	Estimate	Std. error	Z-value	*p*-value	95% CI
Intercept	27.672	0.076	363.36	<0.001	[27.523, 27.821]
VIS_MO (months)	−0.027	0.004	−6.56	<0.001	[−0.034, −0.019]
Random intercept variance (RID)	7.358	0.0749			

## 5 Discussion

Alzheimer’s disease progression remains difficult to predict over short time horizons because clinical trajectories are heterogeneous, progression events are rare, and longitudinal biomarker availability is often incomplete. In this study, we evaluated whether widely available baseline demographic, clinical, and cognitive variables could predict strict 24-month diagnostic progression in ADNI under extreme real-world class imbalance. Our findings demonstrate that baseline cognitive severity and diagnostic status contain measurable predictive signal but also highlight substantial methodological and translational limitations associated with rare-event prediction.

Under the strict 24-month progression definition, only 13 of 2,423 participants (0.5%) met criteria for unidirectional worsening. Despite this extreme imbalance, XGBoost achieved strong rank-based discrimination (AUROC = 0.912), while logistic regression achieved moderate discrimination (AUROC = 0.787). However, precision-recall performance remained low in absolute terms (AUPRC = 0.051), reflecting the mathematical difficulty of predicting rare progression events. Although performance exceeded baseline prevalence (0.5%), these results should not be interpreted as evidence of clinical readiness, as the majority of predicted high-risk cases would represent false positives under real-world deployment conditions (Saito and Rehmsmeier, 2015).

Threshold optimization analyses further highlighted these translational limitations. Even under optimized thresholds, false-positive rates remained high (e.g., 39 false positives for XGBoost and 42 for logistic regression), reinforcing that current models are not suitable for direct clinical deployment. Instead, these findings should be interpreted as an early-stage proof-of-concept demonstrating that baseline clinical features contain detectable signal for short-term progression risk, while underscoring the challenges of translating rare-event prediction models into real-world care settings (Van Calster et al., 2019).

Additional robustness analyses demonstrated that performance was not substantially improved by alternative methodological approaches. Cost-sensitive learning produced results comparable to the primary models, suggesting that algorithm-level weighting alone could not overcome extreme event scarcity. Similarly, alternative imputation strategies yielded mixed results: KNN imputation reduced performance, while MICE produced performance comparable to the primary median-imputed XGBoost model. These findings suggest that the primary limitation of this study was not model selection or preprocessing strategy, but the extremely small number of progression events available for learning and evaluation.

Importantly, logistic regression performed comparably to XGBoost when trained on identical baseline variables, suggesting that complex nonlinear models offered limited incremental benefit. This indicates that much of the predictive signal resides in structured cognitive and diagnostic assessments rather than sophisticated algorithmic architectures. These findings align with broader evidence that simpler models often perform similarly to more complex machine-learning systems in structured clinical datasets when feature engineering is clinically meaningful (Varoquaux and Cheplygina, 2022).

Interpretability analyses further supported the biological plausibility of the models. Across feature importance and SHAP analyses, baseline cognitive severity, functional status, and diagnostic stage consistently emerged as dominant predictors of progression risk. These findings align with prior Alzheimer’s disease literature showing that cognitive impairment severity remains one of the strongest predictors of near-term progression (Yi et al., 2023). Importantly, plasma biomarker and exploratory omics variables contributed minimally to performance in the present study, largely due to incomplete coverage rather than confirmed lack of biological relevance (Schindler et al., 2019; Thijssen et al., 2020).

Longitudinal mixed-effects modeling independently confirmed statistically significant cognitive decline over time (*β* = −0.027 points per month, *p* < 0.001) alongside substantial inter-individual heterogeneity. However, longitudinal slope-derived features were unavailable for most participants (>99% missing in the strict progression cohort) and were therefore excluded from the predictive models. This ensured temporal validity by restricting prediction inputs to baseline variables available at the point of risk estimation.

## 6 Limitations and future work

Several limitations warrant consideration. The extremely small number of progression events within the 24-month window (*n* = 13) substantially limited statistical power and contributed to instability in threshold-dependent performance estimates despite the use of stratified cross-validation. Stratified five-fold cross-validation resulted in validation folds containing only two to three progression events each, meaning that misclassification of a single case could materially alter sensitivity and precision estimates. Although rank-based discrimination remained strong, the high false-positive burden observed during threshold optimization currently limits real-world clinical applicability. In addition, all analyses were conducted exclusively within the ADNI cohort using internal cross-validation, and external validation in independent datasets remains necessary to establish model transportability and generalizability (Riley et al., 2016). Multimodal analyses were further constrained by incomplete plasma biomarker and miRNA coverage, limiting robust evaluation of biological feature integration and requiring cautious interpretation of exploratory findings. Finally, while SHAP improved model transparency by identifying influential predictors, these explanations reflect predictive associations rather than causal mechanisms (Lundberg and Lee, 2017). Future research should prioritize larger event cohorts, longer follow-up windows, external validation across independent populations, and more standardized biomarker collection to determine whether predictive performance can be improved while maintaining interpretability.

## 7 Conclusion

Under a strict 24-month progression definition and extreme class imbalance, baseline demographic, clinical, and cognitive features demonstrated measurable but limited predictive signal for short-term Alzheimer’s disease progression. XGBoost achieved strong discrimination, but low precision and high false-positive rates limit immediate clinical applicability. More complex modeling approaches did not substantially outperform simpler models, and sparse multimodal biomarkers provided minimal incremental benefit in this dataset. These findings should be interpreted as an early-stage proof-of-concept rather than a deployable clinical tool, highlighting both the potential and limitations of machine learning for short-term Alzheimer’s disease progression prediction.

## Data Availability

Publicly available datasets were analyzed in this study. This data can be found at: accessing the data requires an approval. However, the repository can be found at: https://adni.loni.usc.edu/.

## Footnotes

1
nature.com
