# Supplementary material for: Machine learning for Alzheimer’s disease progression under extreme class imbalance
Source: Front Neurosci. 2026 May 25;20:1736992. doi: 10.3389/fnins.2026.1736992 (PMC13243214; doi:10.3389/fnins.2026.1736992)
Supplement: Supplementary file 1 [file Table_1.DOCX]

**4.4 Exploratory Multimodal Analysis**

To explore whether cross-omic relationships modified genetic risk, we evaluated the interaction between APOE ε4 carrier status and the miRNA-Proteome Coupling Index (MPCI) in relation to annual cognitive decline (MMSCORE_SLOPE_YR) in participants with overlapping plasma and miRNA data.

Because sufficient multimodal overlap was available for only 52 participants, these analyses were considered exploratory and were not included in the primary predictive framework. In this subset, APOE ε4 carrier status was associated with steeper cognitive decline (β = -1.78, p = 0.035), while MPCI alone was not independently associated with decline (β = -0.06, p = 0.959). The APOE ε4 × MPCI interaction term was statistically significant (β = 3.73, p = 0.009), suggesting possible genotype-dependent modulation of cross-omic relationships.

Given the limited sample size and high risk of overinterpretation, these findings should be viewed as hypothesis-generating only and are reported in the Supplementary Materials rather than being used to support primary conclusions regarding progression prediction.

**Supplementary Table S1. Exploratory APOE × MPCI Interaction Analysis**

| Predictor | Coefficient | Std. Error | t | p-value | 95% CI |
| --- | --- | --- | --- | --- | --- |
| Intercept | 0.011 | 0.667 | 0.017 | 0.987 | [-1.297, 1.319] |
| APOE ε4 Carrier | -1.782 | 0.844 | -2.112 | 0.035 | [-3.436, -0.127] |
| MPCI | -0.058 | 1.140 | -0.051 | 0.959 | [-2.294, 2.177] |
| APOE ε4 × MPCI | 3.734 | 1.436 | 2.601 | 0.009 | [0.917, 6.550] |
